# Palpable Abdominal Mass is a Renal Oncocytoma: Not All Large Renal Masses are Malignant

**DOI:** 10.1155/2019/6016870

**Published:** 2019-08-08

**Authors:** Sumi Dey, Sabrina L. Noyes, Ghayas Uddin, Brian R. Lane

**Affiliations:** ^1^Spectrum Health, Department of Urology, Grand Rapids, MI, USA; ^2^Spectrum Health, Department of Pathology, MI, USA; ^3^Michigan State University College of Human Medicine, Grand Rapids, MI, USA

## Abstract

A 59-year-old woman presented with abdominal pain and a palpable abdominal mass. Initial imaging revealed a 14cm solid, enhancing renal mass and suspicion for liver and bone metastases. Family history included a brother with clear cell renal cell carcinoma and mother with glioblastoma multiforme. After liver biopsy was inconclusive, she underwent radical nephrectomy with final pathologic diagnosis of oncocytoma. Renal oncocytoma is the most common benign renal tumor but remains difficult to distinguish clinically and radiographically from renal cell carcinoma. Should urologists use renal mass biopsy even more frequently prior to surgical intervention?

## 1. Introduction

Renal oncocytoma is the most common benign renal neoplasm [1, 2], accounting for 3% to 7% of kidney tumors and more common in men than women [3]. The radiographic features of oncocytoma on all forms of conventional imaging are similar to renal cell carcinoma (RCC), making the differential diagnosis particularly challenging [1]. In this case report, we present a patient with family history of RCC and a 14 cm solid, enhancing renal mass with clinical suspicion for metastatic disease that was treated as a malignancy, but found to be an oncocytoma at final pathology.

## 2. Case Presentation

A 59-year-old woman had a palpable mass in the right midabdomen during routine physical examination by her primary care physician. Other than abdominal pain, she had no signs or symptoms of urogenital disorders, such as hematuria or irritative voiding symptoms. She had no prior history of renal disease or trauma. Family history included a brother who underwent nephrectomy for RCC at age 62.

**Figure 1:**
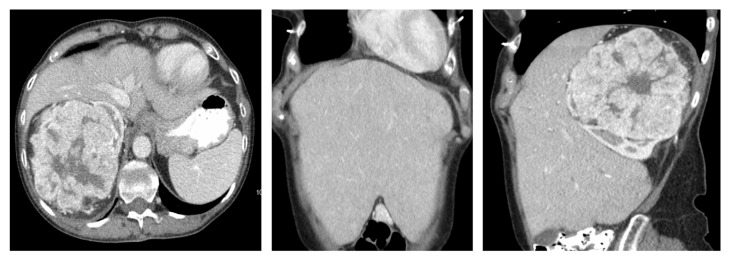
Abdominopelvic CT with IV contrast demonstrates a 14 cm enhancing mass in the right kidney. (a) Axial image shows there was significant enhancement of the solid components of the tumor with finger-like extensions of absent enhancement consistent with necrosis or inflammation. (b) Coronal image demonstrates the enlarged liver that filled all four abdominal quadrants. (c) In sagittal view, the liver is seen to be anteriorly displaced by the large renal mass and to extend down to the right iliac crest.

An abdominopelvic computed tomography (CT) scan revealed a large, solid, right renal mass (14x13x12 cm) and several hypoenhancing liver lesions suspicious for metastases. CT also identified a focal, slightly lucent lesion with sclerotic rim in the T10 vertebral body which was felt to represent a small hemangioma of bone or metastatic lesion, but a subsequent bone scan was negative. CT thorax revealed no metastases. There was significant enhancement of the solid components of the tumor and finger-like extensions of absent enhancement throughout the renal mass consistent with areas of necrosis or acute inflammation (Figure 1(a)). The mass arose from the mid and upper pole of the right kidney and extended superiorly displacing the right lobe of the liver and inferior vena cava (IVC) anteriorly, making the liver palpable (Figure 1(b)). The liver, in fact, extended down to her right iliac crest and below her umbilicus on both sides of her abdomen (Figure 1(c)). There was no evidence of tumor thrombus in the right renal vein or IVC. A diagnosis of likely malignancy with potential liver metastases was made and she was scheduled with interventional radiology for biopsy for pathologic confirmation.

**Figure 2:**
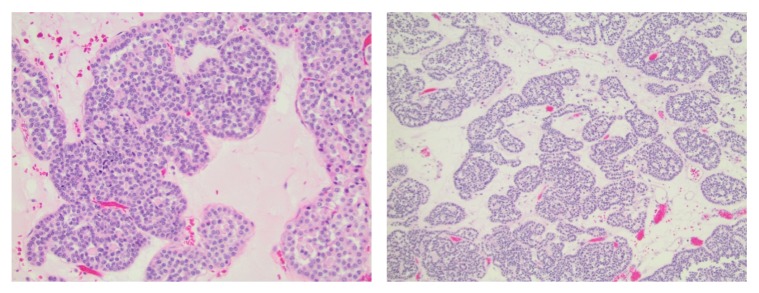
Histological findings of oncocytoma with large eosinophilic cells arranged in distinct nests.

CT scan revealed multiple indeterminate lesions on her liver, the largest of which was 12x10 mm. She was referred for percutaneous biopsy of the kidney and liver lesions for tissue pathology and staging. As well-defined liver lesions were identified, the interventional radiologist performed liver mass biopsy only based on the perceived additional risk of biopsy of the hypervascular renal tumor. Pathology revealed liver parenchyma with fatty deposits, negative for malignancy. Given the negative liver biopsies and normal bone scan, an open radical nephrectomy with right retroperitoneal lymph node dissection was performed for suspected localized RCC using a subcostal incision. With a fixed retractor for the bowel and manual retraction of the liver, exposure of the renal hilum was quite good. No blood transfusion was necessary (estimated blood loss = 100 ml) and the patient was discharged home on postoperative day four without complication. According to final pathologic analysis, including a panel of immunochemical stains which excluded RCC and metanephric adenoma, the diagnosis was renal oncocytoma (Figure 2). Preoperative glomerular filtration rate (GFR) was 101 ml/min/1.73m^2^ and new baseline GFR was 58 ml/min/1.73m^2^. The liver and bone lesions identified prior to surgery have been stable and no local or distant metastases have been seen now nearly four years postoperatively.

## 3. Discussion

Renal oncocytomas were first described in 1942 and the first large series of 13 cases appeared in 1976 from Klein and Valensi [9]. Oncocytoma may coexist with primary RCC, most commonly clear cell and chromophobe RCC. The distinction between oncocytoma and RCC is difficult based on imaging features and generally requires histopathological analysis with a panel of immunochemical stains [10–12]. Oncocytoma has been associated with multiple chromosomal abnormalities including monosomy, trisomy, and loss of heterozygosity [13]. Though benign, renal oncocytomas can grow to very large size with associated symptoms (Table 1). Despite being benign in nature, recent studies have mentioned oncocytic cells integrating with perinephric fat [14, 15] and in rare cases, oncocytoma can invade the renal vein or vein branches [16]. They are generally well-encapsulated and are rarely invasive or associated with metastases [17]. In our case, nephrectomy was indicated for palliation of her abdominal pain and prevention of bleeding from this hypervascular tumor. The presence of only benign histology after surgery indicates that surgery may have been potentially avoided by a renal mass biopsy, but our opinion is that no change in management would have occurred given its large size and the potential of misclassification of an oncocytic RCC because of sampling error [18]. This patient's family history of RCC contributed to our suspicion of RCC, but the specific history in this patient's family was not typical for any known syndrome given the diagnosis of clear cell RCC in a first-degree relative in his 50s and another first-degree relative with a cancer type not known to be associated with any RCC syndromes (glioblastoma multiforme) [19–21]. In this case, genetic evaluation was pursued but did not identify a deleterious mutation in any potentially causative genes. Our plan is to continue follow-up annually with this patient as there have been rare reports of malignant transformation of oncocytoma and she is at moderate risk for progression of CKD [22]. For this reason, surveillance for patients with a histologic diagnosis of oncocytoma from biopsy has been recommended as well [23, 24].

## 4. Conclusion

Renal oncocytoma has a benign clinical course with excellent long term outcomes. Unfortunately, most renal oncocytomas cannot be differentiated from malignant RCC by clinical or radiographic criteria. For this reason, renal mass biopsy is often recommended for small renal masses, as the result may lead such patients to choose surveillance over definitive treatment. For larger masses, such as this report of the sixth largest oncocytoma, radical nephrectomy will remain the preferred management strategy. When metastatic cancer is suspected, biopsy of a metastatic lesion is commonly performed. The primary tumor is occasionally biopsied, but often omitted as malignancy is assumed and the histology of the metastatic lesion establishes the diagnosis and stage. Even in the setting of presumed metastases, renal mass biopsy may be of value because the histologies may not match and the best management strategy may occasionally be altered.

## Figures and Tables

**Table 1:** Largest reported renal oncocytomas.

References	Age (years)	Sex	Tumor size (cm)	Presenting Complaint	Treatment	Metastasis/Recurrences
Demos et al. [4]	64	Male	27x20x15	Palpable abdominal mass	Right open radical nephrectomy	None found

Ahmad et al. [5]	61	Male	25x16x16	Lower back/ flank pain	Right open radical nephrectomy	None found

Banks et al. [6]	57	Male	21x18x15	Abdominal discomfort and distension	Right open radical nephrectomy	1.7 cm left lung nodule and several indeterminate liver lesions at diagnosis. Metastasis could not be excluded.

Kilic et al. [7]	65	Male	20x15x10	Abdominal pain	Left open radical nephrectomy	None found

Sundararajan et al. [8]	37	Male	20	Abdominal mass and moderate hypertension	Right open radical nephrectomy	On follow up at 3 months hypertension had resolved.

Current case	59	Female	14x13x12	Palpable mass	Right open radical nephrectomy	Multiple indeterminate liver, bone and lung lesions at diagnosis with benign liver biopsy
